# The complete mitochondrial genome of the hybrid of Jersey cattle (*Bos taurus*; ♂) × Gannan yak (*Bos grunniens*; ♀)

**DOI:** 10.1080/23802359.2019.1692721

**Published:** 2019-11-20

**Authors:** Xian Guo, Pengjia Bao, Xiaoyun Wu, Zhen Yang, Shengguang Shi, Lin Xiong, Jie Pei, Ping Yan

**Affiliations:** a Key Laboratory of Yak Breeding Engineering of Gansu Province, Lanzhou Institute of Husbandry and Pharmaceutical Sciences, Chinese Academy of Agricultural Sciences, Lanzhou, Gansu, People’s Republic of China;; b Animal Husbandry Station of Hezuo City, Hezuo, Gansu, People’s Republic of China

**Keywords:** Bayesian inference, high-throughput sequencing, interspecific hybridization, iterative mapping, mitogenomics

## Abstract

In this study, we reconstructed the complete mitochondrial genome of the hybrid of Jersey cattle (*Bos taurus*; ♂) × Gannan yak (*Bos grunniens*; ♀) from Illumina sequencing reads. The mitochondrial genome is 16,322 bp in length with an A + T-biased nucleotide composition, and encodes 13 protein-coding genes, 22 tRNAs, and 2 rRNAs along with a noncoding control region. In addition, its gene order is identical to those of previously published mitochondrial genomes of the genera *Bison* and *Bos*. Phylogenetic analysis indicates that this hybrid is most closely related to Gannan yak and Jinchuan yak.

Interspecific hybridization has long been employed by humans to produce hybrid offspring which are sometimes stronger or perform better than either parental lineage (i.e. hybrid vigor or heterozygote advantage). An insight into their genetics and genomics would facilitate the development and exploitation of such resources. In this study, we reconstructed the complete mitochondrial genome of the hybrid of Jersey cattle (*Bos taurus*; ♂) × Gannan yak (*Bos grunniens*; ♀) from Illumina sequencing reads. This hybrid has been locally maintained for milk (female) and meat (male) in Hezuo City, Gannan Tibetan Autonomous Prefecture, Gansu Province, China, and is well-adapted to the local high-altitude, cold, and anoxic environment.

A blood sample was collected from Qinaihe Village, Kajiadao Township, Hezuo City (35°11′N, 103°00′E). A voucher specimen is held in the Key Laboratory of Yak Breeding Engineering of Gansu Province, Lanzhou Institute of Husbandry and Pharmaceutical Sciences (Lanzhou, Gansu Province, China). The genomic DNA coded as No. 20190919, which was extracted from Jersey cattle (*B. taurus*; ♂) × Gannan yak (*B. grunniens*; ♀) is stored at −80 °C (ultra deep-freeze refrigerator) in the sample storage room of our department. Genomic DNA extraction with the QIAamp DNA Blood Mini Kit (Qiagen, Orinda, CA), library preparation and high-throughput sequencing with Illumina HiSeq X™ Ten Sequencing System (Illumina, San Diego, CA) were conducted by Annoroad Gene Technology (Beijing, China). The resultant sequencing reads were then used to reconstruct the mitochondrial genome with MITObim v1.9 (Hahn et al. 2013); the reference sequence (GenBank accession: JQ692071) was retrieved from a previous study by Qiu et al. (2012). The mitochondrial genome was annotated by aligning with those of its congeners.

The mitochondrial genome of the hybrid (GenBank accession: MN163007) is 16,322 bp in length with an A + T-biased nucleotide composition (33.7% A, 25.9% C, 13.2% G, & 27.2% T; ‘light strand’). As found in most animals, it encodes 13 protein-coding genes (PCGs), 22 tRNAs and two rRNAs along with a noncoding control region. Its gene order is identical to those of the previously published mitochondrial genomes of the genera *Bison* and *Bos* (e.g. Douglas et al. 2011; Wu et al. 2016, 2018). Two types of start codons (ATA & ATG) and three types of stop codons (TAA, TAG & T) were annotated for all 13 PCGs. The 22 tRNAs range in length from 60 bp (*tRNA-Ser^AGN^*) to 75 bp (*tRNA-Leu^UUR^*) with a total length of 1509 bp. The two rRNAs are 957 bp (*12S rRNA*) and 1571 bp (*16S rRNA*) long, respectively. The control region is 895 bp long with an A + T content of 61.4%.

A Bayesian tree was reconstructed to investigate its relationship with 24 taxa within the genera *Bos* and *Bison* using the program MrBayes v3.1.1 (Ronquist and Huelsenbeck 2003) as implemented in TOPALi v2.5 (Milne et al. 2009). All 13 PCGs were used for the phylogenetic analysis, and ‘GTR + G’ was employed as the best-fit nucleotide substitution model (Figure 1). The hybrid was found to be most closely related to Gannan yak (Wu et al. 2016) and Jinchuan yak (Mipam et al. 2012). In addition, our study also indicated that the interrelationship between the two genera *Bison* and *Bos* may need further investigations.

**Figure 1.**
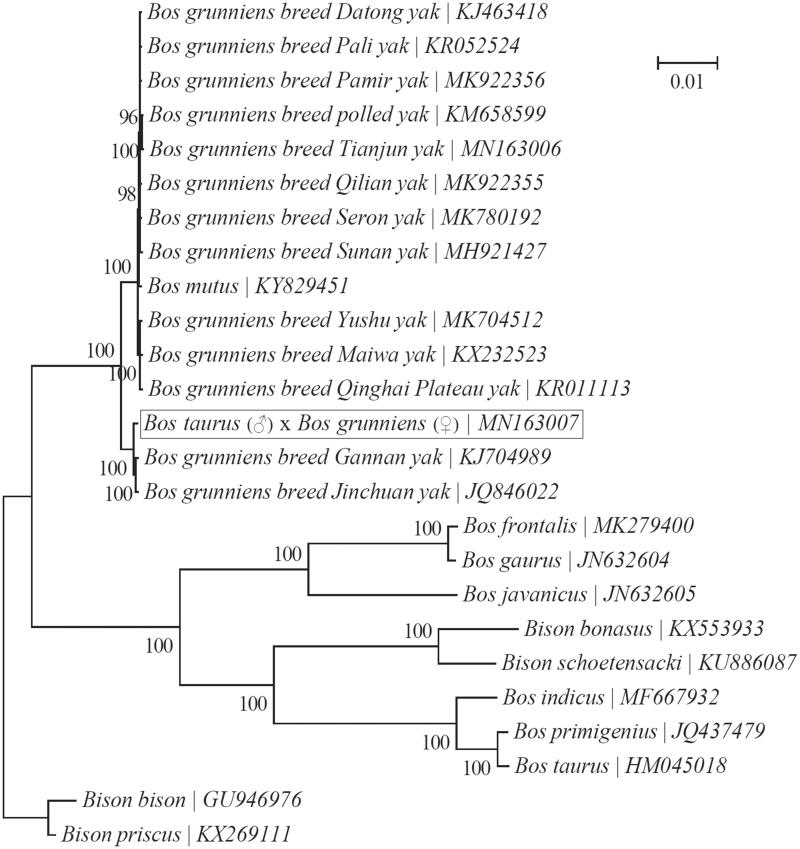
Phylogeny of two related genera *Bison* and *Bos* based on the Bayesian analysis of the concatenated sequences of 13 mitochondrial protein-coding genes (alignment size: 11,370 bp). The best-fit nucleotide substitution model is ‘GTR + G’.
